# Causal Forests in Practice: Lessons on Detecting Heterogeneous Treatment Effects in a Randomized Controlled Trial of a Healthy Food Subsidy Program in Canada

**DOI:** 10.1016/j.tjnut.2026.101529

**Published:** 2026-04-08

**Authors:** Michelle L Aktary, Inara Lalani, Yong Chen, Zahra Shakeri, Gavin R McCormack, Sharlette Dunn, Tolulope Sajobi, Heather O'Hara, Peter Leblanc, Jenny Godley, Natalie Doan, Dana Lee Olstad

**Affiliations:** 1 Faculty of Kinesiology, University of Calgary, Calgary, AB, Canada; 2 Department of Community Health Sciences, Cumming School of Medicine, University of Calgary, Calgary, AB, Canada; 3 Institute of Health Policy, Management and Evaluation and Dalla Lana School of Public Health, University of Toronto, Toronto, ON, Canada; 4 British Columbia Association of Farmers' Markets, Vancouver, BC, Canada; 5 Department of Anthropology and Archaeology, University of Calgary, Calgary, AB, Canada; 6 School of Public Health Sciences, University of Waterloo, Waterloo, ON, Canada

**Keywords:** diet quality, farmers’ market healthy food subsidy, heterogeneous treatment effects, randomized controlled trial, machine learning

## Abstract

**Background:**

There is limited guidance on the use of causal forests in moderately sized randomized controlled trials (RCTs).

**Objectives:**

This study aimed to apply and evaluate a causal forest to estimate heterogeneous treatment effects of a healthy food subsidy program using data from a moderately sized RCT.

**Methods:**

Using data from an RCT of the British Columbia Farmers’ Market Nutrition Coupon Program (FMNCP) (*n* = 263), a causal forest analysis examined heterogeneous treatment effects on Healthy Eating Index-2015 scores (HEI-2015; 0–100) postintervention. Treatment effect heterogeneity was assessed in the following 3 ways: *1*) using the best linear prediction test to measure covariance between predicted and true treatment effects, *2*) comparisons of high versus low treatment effect groups, and *3*) estimating rank-weighted average treatment effects on a targeting operator characteristics (TOC) curve and estimating the area under the TOC (AUTOC). A simulation-based power analysis examined the sample size at which the causal forest could detect a 5-point difference in HEI-2015 scores between participants with high compared with low educational attainment.

**Results:**

The best linear prediction test showed poor calibration and did not detect heterogeneous treatment effects (*β* = 0.44, *P* = 0.27). Heterogeneity was not detected when comparing groups with high compared with low treatment effects [–2.25, 95% confidence interval (CI): –13.53, 9.03]. The TOC curve was flat with wide CIs, and the AUTOC was not significant (–1.02; *P* = 0.77). Based on the simulation-based power analysis, 1050 participants were required to achieve 80% power, whereas our sample provided 40% power.

**Conclusions:**

A causal forest did not detect heterogeneous treatment effects of the FMNCP on the diet quality of adults with low incomes. Simulation results indicated that the trial was underpowered, underscoring the need for larger trials. Nevertheless, with careful application and evaluation, causal forests remain a useful tool to explore heterogeneous treatment effects in moderately sized trials when the sample size is adequate.

This trial was registered at clinicaltrials.gov as NCT03952338 (https://clinicaltrials.gov/ct2/home).

## Introduction

Consuming a high-quality diet is essential to reduce the risk of nutrition-related chronic diseases [1,2]. In Canada and other high-income countries, inequities in diet quality have contributed to a disproportionately high burden of poor health outcomes among populations with low incomes [[3], [4], [5], [6], [7]]. Among the many determinants of diet quality, the higher cost of nutrient-dense foods, such as fruits and vegetables, is an especially formidable barrier to maintaining a healthy dietary pattern [[8], [9], [10], [11], [12]].

Farmers’ markets healthy food subsidy programs have become a popular strategy for reducing financial and access-related barriers to consuming healthy foods among populations with low incomes [13,14]. In Canada, the British Columbia Farmers’ Market Nutrition Coupon Program (BC FMNCP) provides low-income households with coupons to purchase healthy, locally grown foods from BC farmers’ markets [15]. In 2019, we conducted a parallel-group pragmatic randomized controlled trial (RCT) to investigate the impact of the BC FMNCP on the diet quality, household food insecurity, and psychosocial well-being of adults with low incomes [[16], [17], [18]]. Although the FMNCP reduced short-term household food insecurity by 79%, there were no significant between-group differences in diet quality [Healthy Eating Index (HEI)-2015 scores] overall or according to age group or sex [17]. However, program impacts may have varied across other participant subgroups; a concept referred to as heterogeneous treatment effects [19,20].

Precision public health “investigates how multiple dimensions of social position interact to confer health risk differently for precisely defined population subgroups according to the social contexts in which they are embedded” [21]. Within a precision public health framework [21], identifying heterogeneous treatment effects of healthy food subsidy programs can provide valuable insights into which subgroups benefit most. Furthermore, heterogeneous treatment effect estimation can reveal unintended negative consequences, such as increases in dietary inequities (e.g., if participants with higher incomes benefited more from the program than those with lower incomes) [22].

Several observational studies have examined heterogeneous treatment effects of fruit and vegetable subsidies on fruit and vegetable intake using regression-based methods and found no evidence of heterogeneity [23,24]. Two studies using data from an RCT of a healthy food subsidy program (*n* = 332) also produced mixed findings [25,26]. One study applied quantile multivariate regression methods and found that changes in fruit and vegetable intake varied by baseline fruit and vegetable intake and household size [26]. The other study used matching with classification and regression trees and found no treatment effect heterogeneity in fruit and vegetable intake or diet quality [25]. Together, these studies suggest that evidence pertaining to the differential impacts of healthy food subsidy programs remains limited.

Traditional approaches to examining treatment effect heterogeneity, such as regression models with interaction terms [27,28], are statistically valid and produce interpretable results when examining a single effect modifier [29]. However, these approaches are constrained by the number and functional form of predictors that can be examined simultaneously, low statistical power and overfitting, and increased risk of false positives from multiple testing [27,30]. Moreover, these approaches require prespecifying potential effect modifiers, which can limit the discovery of novel factors influencing treatment effect heterogeneity [27,30].

To overcome some limitations of these conventional approaches, Athey et al. [20,31,32] developed nonparametric machine learning methods to estimate heterogeneous treatment effects using causal forests. Similar to random forest algorithms [33], causal forests have the potential to address these limitations by using data-driven procedures to construct a collection of individual decision trees to predict outcomes [19,20]. However, there are several key distinctions that make causal forests better suited to identify heterogeneous treatment effects than regression-based analyses and other machine learning approaches. First, they are causal in that they predict treatment effects (i.e., the difference between the expected outcome if an individual were in the treatment compared with the control group) rather than the outcome itself (i.e., conditional mean estimation) [19,20]. Second, causal forests employ honest estimation, such that one subsample is used to construct the causal tree and for cross-validation, whereas a separate subsample is used to estimate treatment effects within each leaf [19,20]. Honest estimation reduces bias and overfitting and facilitates causal inference [19,20]. Third, causal forests use a data-driven approach to identify complex functional forms such as nonlinear relationships and higher-order interactions among variables, without explicitly prespecifying interaction terms [20,34]. Finally, causal forests are less susceptible to overfitting, perform well with smaller sample sizes and many covariates, and can more readily handle missing data [20,35].

Despite their theoretical advantages, there is limited guidance on how causal forests can be applied, evaluated, and interpreted in moderately sized RCTs, where sample size constraints can reduce the power to detect treatment effect heterogeneity and increase the variance and uncertainty of effect estimates [36,37]. Accordingly, this study aimed to apply and evaluate causal forests to estimate heterogeneous treatment effects in a moderately sized RCT of a healthy food subsidy program.

The objectives of this study were 2-fold: *1*) to conduct an exploratory causal forest analysis to examine heterogeneous treatment effects of the FMNCP on the diet quality of adults with low incomes, and *2*) to conduct a simulation-based power analysis to assess whether the sample size was sufficient to detect simulated heterogeneous treatment effects in the FMNCP.

## Methods

### Study design and participants

We conducted an exploratory analysis of data from a parallel-group pragmatic RCT undertaken in 2019 that examined the impact of the FMNCP on the diet quality (primary outcome), household food insecurity, and psychosocial well-being of adults with low incomes [[16], [17], [18]]. The RCT is briefly described below, with further details available elsewhere [[16], [17], [18]].

Community partner organizations, such as pregnancy outreach agencies, recruited study participants from among their existing clients who were on the FMNCP waitlist either in person, by telephone, or by email. Adults eligible for the RCT were aged ≥18 y, considered to have low incomes based on FMNCP income cut-offs (e.g., often <CAD $18,000/y, but cut-offs were higher in communities with a higher cost of living), had not participated in the FMNCP in previous years, lived with ≤8 people including the participant, were the primary food shopper, did not self-report dementia or Alzheimer's disease, could communicate in English (or had assistance), and did not anticipate relocating or changes in household income or composition during the study.

Participants were randomly assigned 1:1 to an FMNCP intervention (*n =* 143) or a no-intervention control group (*n =* 142). Participants in the FMNCP group received 16 wk’ worth of coupons valued at CAD $21/wk (the subsidy amount provided by the program in 2019) over 10–15 wk to purchase fruits and vegetables, dairy, meat, poultry, fish, eggs, nuts, and cut herbs at BC farmers’ markets. Those in the FMNCP group were also invited, but not required, to attend nutrition skill-building activities, such as cooking classes. Participants in the control group did not receive coupons nor participate in nutrition skill-building activities.

The RCT was conducted in accordance with procedures outlined in the Tri-Council Policy Statement and the Declaration of Helsinki. Ethical approval was obtained from the Conjoint Health Research Ethics Board at the University of Calgary (REB18-0508), University Ethics and Compliance at Rutgers University (FWA00003913), and the Office of Research Ethics at the University of Waterloo (ORE #40724). Eligible participants provided voluntary informed consent to participate in the study before completing baseline data collection. The current study is described in accordance with the Strengthening the Reporting of Observational Studies in Epidemiology Statement [38].

### Data sources

Participants completed an online questionnaire that collected self-reported sociodemographic characteristics and health-related information, and two 24-h dietary recalls at baseline (week 0; May–August 2019), after intervention (weeks 10–15; October–November 2019), and 16 wk after intervention (weeks 26–31; February–March 2020). The current study used data from baseline and postintervention only.

### Outcome: HEI-2015 scores

The primary outcome of this study was diet quality (HEI-2015 scores) postintervention and did not change during the course of the study. Dietary intake data were collected using the Automated Self-Administered 24-h Dietary Recall for Canada (ASA24-Canada-2018), an online automated dietary assessment tool [39]. The ASA24-Canada-2018 was adapted from the ASA24 to reflect the Canadian food supply [40]. Participants recorded all foods and beverages consumed from midnight to midnight the previous day. Participants were invited to complete a second dietary recall 2–5 d later [41].

Diet quality was calculated using the validated HEI-2015, a tool used to assess alignment with the 2015–2020 Dietary Guidelines for Americans [42]. Although a Canadian-adapted version of the HEI-2015 (the HEI-2019) exists, it was not released until after the RCT was preregistered [43]. Nonetheless, the HEI-2015 is one of the most robust diet quality indices and is recommended for use in international studies for this reason [44]. HEI-2015 total scores can range from 0 to 100, with higher scores indicating higher diet quality [45]. To calculate HEI-2015 total scores, dietary intake data were coded using the Canadian Nutrient File (2015 version), the United States Food and Nutrient Database for Dietary Studies [46], and the USDA’s Food Patterns Equivalents Database [47] to estimate food and nutrient intakes required to calculate subscores for adequacy (total fruits, whole fruits, total vegetables, greens and beans, whole grains, dairy, total protein foods, seafood and plant proteins, and fatty acids) and moderation (refined grains, sodium, added sugars, and saturated fats) components in the HEI-2015 [48]. Subscores for adequacy and moderation components were then summed using SAS macros from the National Cancer Institute [45].

The study sample used for the causal forest analysis included only those who completed one or both dietary recalls after intervention. HEI-2015 scores were averaged for those who completed 2 dietary recalls to better estimate the mean usual intake [41,49].

### Predictors

Baseline candidate predictor variables were selected based on the following: *1*) the most important variables identified in a preliminary causal forest [32,50,51] (Supplemental Table 1), *2*) Pearson correlation coefficients between baseline predictors and HEI-2015 scores postintervention (Supplemental Table 2), and *3*) sociodemographic characteristics and health practices associated with diet quality identified in the published literature (Supplemental Table 3). On the basis of these 3 considerations, a total of 12 baseline variables were selected for the causal forest including BMI (continuous), HEI-2015 scores (continuous), years lived in Canada (continuous), annual household income before taxes (<$20,000, $20,000–$39,999, $40,000–$59,999, and ≥$60,000 CAD), age (continuous), sex (male or female), marital status (living with partner or not living with partner), children living in the home (yes or no), educational attainment (high school diploma or less, some postsecondary or trade, Bachelor's degree, and Graduate degree), geographic location (urban or rural), race/ethnicity (White or racial/ethnic minority group), and smoking status (yes or no).

Causal forests can only accommodate numerical variables (i.e., continuous and ordered categorical variables) [52]. A common approach to converting nominal variables to numerical variables is one-hot encoding, whereby each level of the variable is converted to a binary variable [19]. However, this method may lead to overfitting and less precise estimates; therefore, nominal variables, such as race/ethnicity, were dichotomized.

### Missing data

Predictors with missing values were handled using the missing incorporated in attributes method implemented natively in the Generalized Random Forest (*grf)* package in R (v 2024.04.2 + 764, R Foundation for Statistical Computing) [[52], [53], [54]]. This approach treats missing values as a separate category for the relevant attribute and assigns them to the side of the split that improves the splitting criterion, independent of where the split occurred [[52], [53], [54]]. This method minimizes error at a similar rate to multiple permutation approaches [54].

### Data analyses

Descriptive statistics were calculated using Stata (v17.0, Stata Corp), and a causal forest was trained using *grf* to examine heterogeneous treatment effects among FMNCP study participants [31]. Following the potential outcomes framework [55], causal forests predict treatment effects as the difference between 2 potential outcomes conditional on treatment or control group assignment [20,31,32]. The conditional average treatment effect (CATE) corresponds to the potential HEI-2015 score if a participant was randomly assigned to the treatment compared with the control group, conditional on baseline predictors [20,31,32]. The average treatment effect (ATE) is the average difference in potential HEI-2015 scores if all participants were assigned to the treatment group compared with the control group [20,31,32].

The causal forest algorithm begins by using a random subset of baseline variables to identify predictors and split points that maximize the heterogeneity of treatment effects and minimize the expected mean squared error of treatment effects [19,20]. These splits continue through all inputted predictors until no further splits can be made (e.g., if there are no valid splits) [52]. Each resulting decision tree estimates the CATE of participating in the FMNCP within its terminal leaves using out-of-bag predictions [19,20,31,32]. The causal forest algorithm protects against imbalances by ensuring an equal number of participants in the intervention and control groups in each leaf [52].

To fit the causal forest, we used default model parameters included in *grf*, which perform reasonably well across diverse use cases and enable internal cross-validation [20,31,32]. We fit a causal forest with 11,000 trees, the number of trees at which the mean variance of treatment effect estimates was minimized (Supplemental Figure 1). Although fewer trees can reduce computational burden, larger forests generally improve the stability of variance estimates and the precision of confidence intervals (CIs) [52]. The cross-validation procedure in grf was enabled, allowing model parameters (e.g., the number of variables tried for each split) to be tuned to improve the precision of estimates [20,31,32]. All observations were used to estimate the ATE [50]. The dataset was then randomly divided into a training set (70% of observations) and a testing set (30% of observations) to estimate and evaluate CATEs. Although there is no precise train/test ratio, a larger training set is recommended with smaller sample sizes [50,56]. Because causal forest performance can be sensitive to training parameters, researchers may wish to explore alternative configurations (e.g., varying the number of trees or sample splitting proportions) and evaluate model performance using methods described below or other diagnostic checks [52]. Model assumptions and fit were evaluated and are detailed in Supplemental Figures 2–4.

Treatment effect heterogeneity was examined using 3 methods, which also provided insight into model calibration and ranking performance. First, the best linear predictor test fits out-of-bag CATE predictions as a linear function of the true CATEs [30,32,57]. The best linear prediction test provides estimates for *α* (mean forest prediction) and the slope *β* (differential forest prediction). An *α* of 1 indicates that the causal forest produced accurate predictions [32,57]. A *β* of 1 indicates the causal forest predictions adequately captured the underlying heterogeneity, along with the *P* value, which provides an omnibus test for the presence of treatment effect heterogeneity [32,57]. Second, using the model developed in the training sample, FMNCP participants in the test set were grouped according to whether their predicted CATEs were above or below the median CATE estimate, and the ATE and 95% CIs were estimated for each subgroup and compared [32]. Third, the rank-weighted ATE prioritizes participants according to their estimated CATES. The prioritization rules give higher scores to participants with larger CATEs and lower scores to those with smaller CATEs, then compute the ATE for each subgroup in this ranking [50,58]. The computed ATEs are compared to the overall ATE and plotted in a targeting operator characteristic (TOC) curve [50,58]. The point estimate of the area under the TOC (AUTOC) and *P* value is then calculated. A positive and significant AUTOC indicates that the causal forest successfully ranked individuals with higher CATEs, consistent with the presence of treatment effect heterogeneity, whereas an AUTOC that is zero or negative indicates that the model did not detect heterogeneity [50,58].

After examining heterogeneous treatment effects, the variable importance measure was generated for each variable. Variable importance weighs variables based on their inclusion and depth within the tree, reflecting their influence in identifying treatment effect heterogeneity [51,59]. Variable importance scores range from 0 to 1, where a higher score indicates a more prominent role in sample splitting [32,50,60]. Variable importance is determined by a variable’s ranking relative to others in the causal forest, rather than an absolute value (e.g., more variables will lead to lower absolute variable importance measures) [32,50,60]. The 6 most important variables’ CATE predictions and SEs were obtained from the causal forest. Continuous variables were grouped into quartiles to enhance interpretability, whereas categorical variables were left unchanged. Mean CATEs and 95% CIs were plotted across categories of each variable.

Finally, as a baseline comparator to the causal forest, linear regression models were fit with a 3-way interaction between the treatment indicator variable (FMNCP compared with control group) and the 2 highest-ranking causal forest variables, and 2-way interactions between the treatment indicator variable and each of these 2 variables. We limited this exploratory assessment of effect modification to 2 variables, given that the main RCT had already examined prespecified effect modifiers (age group and sex).

### Sensitivity analyses

To examine whether excluding participants with missing outcome data from the main analysis influenced the results, a sensitivity analysis was conducted whereby missing HEI-2015 scores after intervention were imputed using multiple imputation by chained equations (MICE). Although we used *grf’s* missing incorporated in attributes method in the main analysis to impute missing covariate values, this method is not designed to handle missing outcome values [54]. Therefore, we used MICE to impute missing outcome and covariate values before running the causal forest to ensure consistency across imputed datasets. Missing data were imputed 5 times, using predictive mean matching for continuous variables, logistic regression for dichotomized variables, and proportional odds logistic regression for ordinal variables. ATEs and estimates from the best linear prediction test were estimated within each imputed dataset and then pooled. In addition, a single imputed dataset was used to plot a TOC curve and estimate the AUTOC.

In a second sensitivity analysis, a causal forest was trained using only the 6 most important variables based on their relative rankings in the main analysis (BMI, baseline HEI-2015 score, age, years lived in Canada, annual household income, and educational attainment) to provide more precise estimates [32,61]. Although there is no standard cutoff for variable importance in causal forests, we selected these predictors to test model robustness while reducing potentially noisy or low-contribution variables. Heterogeneity was tested using the best linear prediction test, plotting a TOC curve, and estimating the AUTOC.

Some categorical variables were dichotomized in the main analysis to reduce dimensionality, which may have resulted in missed heterogeneity. Therefore, to examine whether dichotomizing variables influenced findings, the variable children living in the home (yes or no) was replaced with the number of children in the home (1, 2, 3, and 4+). In addition, a Canadian study found that diet quality was higher among adults from racial/ethnic minority groups but lower among Indigenous adults than White adults [62]. Therefore, race/ethnicity was recorded in 2 ways and included in separate causal forests with the number of children and other predictors included in the main analysis. First, one-hot encoding was used to create binary variables for each race/ethnicity category (i.e., White, East and Southeast Asian, South and West Asian, Indigenous, and all other racial/ethnic groups). Second, Indigenous identity (Indigenous compared with non-Indigenous) and race/ethnicity (White compared with racial/ethnic minority, excluding Indigenous peoples) were treated as separate variables. Heterogeneity was tested using the best linear prediction test, plotting a TOC curve, and estimating the AUTOC.

Finally, household food insecurity is an important predictor of diet quality, with more severe experiences associated with progressively poorer diet quality [[63], [64], [65]]. Therefore, program impacts may have varied according to the severity of household food insecurity. Although we limited our main causal forest analysis to prespecified baseline covariates considered for model adjustments in the FMNCP RCT, we conducted a sensitivity analysis that included baseline severity of household food insecurity, a secondary outcome of the RCT. Household food insecurity was categorized as food secure, and marginal, moderate, and severe food insecurity [16,18]. Heterogeneity was tested using the best linear prediction test, plotting a TOC curve, and estimating the AUTOC.

### Simulation-based power analysis

A simulation-based power analysis was conducted to determine the sample size at which the causal forest could detect treatment effect heterogeneity. A clinically meaningful 5-point between-group difference in HEI-2015 scores [49] was generated for participants with low compared with high educational attainment, a key determinant of diet quality and dietary inequities [66]. Gaussian noise was added to approximate the residual variability observed in the dataset.

Simulated datasets were generated at sample sizes comparable with and larger than the original trial (*n* = 200, 263, 350, 500, 750, 1000, 1250, and 1500). For each sample size, simulated datasets were generated by resampling participants’ baseline covariates from the original FMNCP data. Treatment was assigned using the RCT’s randomization probability of 0.5. Causal forests were fit using default *grf* settings (2000 trees, cross-validation and honesty enabled), and each simulated dataset was randomly split into 70% training and 30% testing sets.

Power to detect the simulated heterogeneity in HEI-2015 scores between high and low educational attainment was estimated as the proportion of simulations (*R* = 200 per sample size) in which the best linear predictor test produced a *P* value <0.05, and results were summarized in a power curve. The minimum sample size required to achieve ≥80% power was then identified. This sample size was subsequently used to examine heterogeneous treatment effects using the best linear predictor test and the TOC curve and corresponding AUTOC.

## Results

In the RCT, 404 adults with low incomes were assessed for eligibility, and 119 were excluded due to ineligibility or nonresponse. Of the 285 participants randomly assigned to the FMNCP (*n =* 143) or control (*n =* 142) group, 13 were lost to follow-up at after intervention, and an additional 9 participants were excluded due to missing HEI-2015 scores. The causal forest analysis included 263 participants (*n =* 137 FMNCP; *n =* 126 control) who completed 1 or both 24-h dietary recalls, representing 92.3% of participants randomly assigned. Participant characteristics were similar to those in the RCT [17] and are summarized in Table 1. Participants were aged on average 43 ± 16 y. Most were female (90.5%), reported being from a racial/ethnic minority group (62.9%), and had children living in the home (65.6%), an annual household income under CAD $20,000/y (39.0%), and a high school education or less (39.9%). Participants had a mean baseline HEI-2015 score of 60.2 ± 14.5 (of 100). Nearly all (94.7%) participants completed 2 dietary recalls and shared similar characteristics to those who completed a single recall.

**TABLE 1:** Baseline characteristics of adults with low incomes (*n* = 263) in the British Columbia Farmers’ Market Nutrition Coupon Program randomized controlled trial.

Characteristics	Control (*n* = 126) *n* (%)	FMNCP (*n* = 137) *n* (%)	Total (*n* = 263) *n* (%)
Sex			
Male	12 (9.5)	13 (9.5)	25 (9.5)
Female	114 (90.5)	124 (90.5)	238 (90.5)
Children living in the home			
Yes	81 (64.3)	91 (66.9)	172 (65.6)
No	45 (35.7)	45 (33.1)	90 (34.4)
Educational attainment			
High school or less	48 (38.7)	55 (41.0)	103 (39.9)
Some postsecondary or trade	36 (29.0)	43 (32.1)	79 (30.6)
Bachelor’s degree	16 (12.9)	25 (18.7)	41 (15.9)
Graduate degree	24 (19.4)	11 (8.2)	35 (13.6)
Race/ethnicity			
White	43 (37.7)	45 (36.6)	88 (37.1)
Black	5 (4.4)	3 (2.4)	8 (3.4)
East Asian	6 (5.3)	11 (8.9)	17 (7.2)
Southeast Asian	4 (3.5)	2 (1.6)	6 (2.5)
South Asian	22 (19.3)	21 (17.1)	43 (18.1)
Arab or West Asian	20 (17.5)	22 (17.9)	42 (17.7)
Indigenous	8 (7.0)	15 (12.2)	23 (9.7)
Latin American	4 (3.5)	3 (2.4)	7 (3.0)
Any other race/ethnicity	2 (1.8)	1 (0.8)	3 (1.3)
Annual household income ($)			
<20,000	45 (40.9)	44 (37.3)	89 (39.0)
20,000–39,999	41 (37.3)	38 (32.2)	79 (34.6)
40,000–59,999	12 (10.9)	22 (18.6)	34 (14.9)
≥60,000	12 (10.9)	14 (11.9)	26 (11.4)
Household food insecurity			
Food secure	34 (28.3)	23 (17.8)	57 (22.9)
Marginal food insecurity	6 (5.0)	14 (10.9)	20 (8.0)
Moderate food insecurity	53 (44.2)	57 (44.2)	110 (44.2)
Severe food insecurity	27 (22.5)	35 (27.1)	62 (24.9)
Marital status			
Not living with partner	50 (40.0)	63 (47.0)	113 (43.6)
Living with partner	75 (60.0)	71 (53.0)	146 (56.4)
Smoking status			
Nonsmoking	110 (87.3)	120 (87.6)	230 (87.5)
Smoking	16 (12.7)	17 (12.4)	33 (12.5)
Geography			
Rural	17 (13.5)	20 (14.6)	37 (14.1)
Urban	109 (86.5)	117 (85.4)	226 (85.9)

	Mean (SD)	Mean (SD)	Mean (SD)

Healthy Eating Index 2015 total score (0–100)	60.1 (14.9)	60.4 (14.1)	60.2 (14.5)
Age (y)	41.9 (16.6)	44.0 (16.1)	43.0 (16.4)
BMI (kg/m^2^)	26.3 (6.4)	27.4 (5.4)	26.8 (5.9)
Years lived in Canada	23.8 (22.5)	25.4 (22.6)	24.6 (22.5)

Abbreviations: FMNCP, Farmers’ Market Nutrition Coupon Program.

Baseline predictor variables with missing values ranged from 0.4% (years lived in Canada and number of children living in the home) to 13.3% (annual household income). There were no significant differences in missingness between groups.

### CATEs and ATEs

**FIGURE 1:**
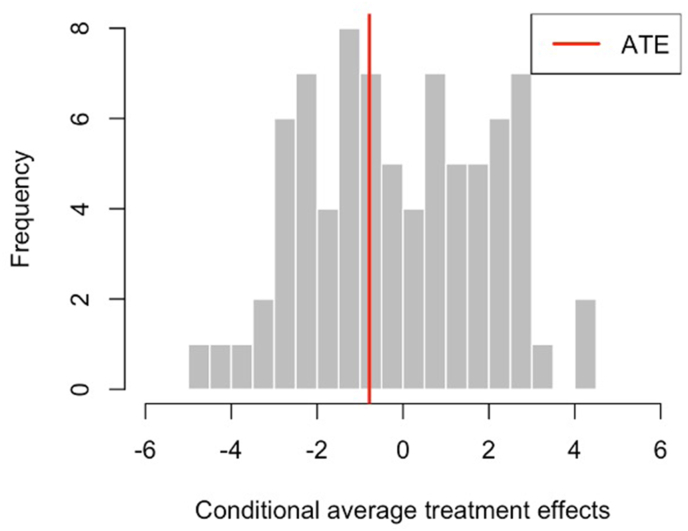
Histogram of average treatment effects (ATE) and conditional average treatment effects.

The distribution of the CATEs and the ATEs is presented in Figure 1. The ATE was –0.78 (95% CI: –3.89, 2.33). Although there was a clear variation in treatment effects around the ATE, the visual distribution of CATEs suggested, but did not necessarily indicate, the presence of treatment effect heterogeneity [32].

### Heterogeneous treatment effects

Results from the best linear predictor test are shown in Table 2. The nonsignificant estimate of *α* (the mean forest prediction) suggests that the causal forest predictions were not well calibrated to the true treatment effects. Additionally, the estimate of *β* (the differential forest prediction) was <1 and the associated *P* value was not significant (*P =* 0.27), suggesting that the causal forest did not detect meaningful variation in treatment effects.

**TABLE 2:** Causal forest best linear predictor estimates.

	Estimate	*P* value
α (mean forest prediction)	0.95	0.31
*β* (differential forest prediction)	0.44	0.27

**FIGURE 2:**
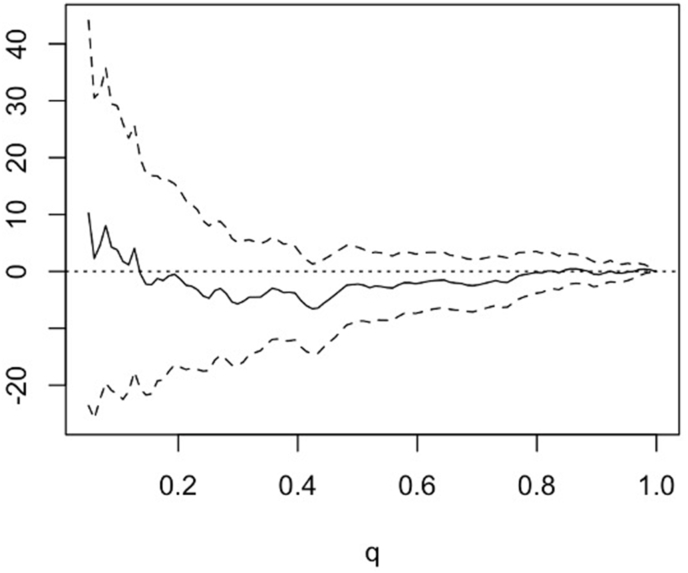
Targeting operator characteristic curve for predicted conditional average treatment effects and pointwise 95% confidence intervals (dashed lines). Note: *q* = fraction treated.

When grouped according to high compared with low predicted CATEs, the between-group difference in ATEs was not significant [–2.25 (95% CI: –13.53, 9.03)]. The negative value and wide CI suggest uncertainty in estimates. Similarly, the TOC curve was relatively flat with wide CIs that overlapped zero (Figure 2). As shown in Figure 2, the *x*-axis is the fraction of individuals treated based on their CATEs (*q*), and the *y*-axis is the ATE of treating the *q*th fraction of individuals minus the ATE of treating the entire sample (*y* = 0) [50]. Values above zero would indicate that the model successfully identified individuals with higher treatment effects; however, estimates remained close to or below zero across all values of *q*. Consistent with this, the AUTOC was negative and not significant [–1.02 (*P =* 0.77)], indicating that the causal forest did not accurately rank participants by their predicted CATEs and did not detect treatment effect heterogeneity.

### Variable importance measures

**FIGURE 3:**
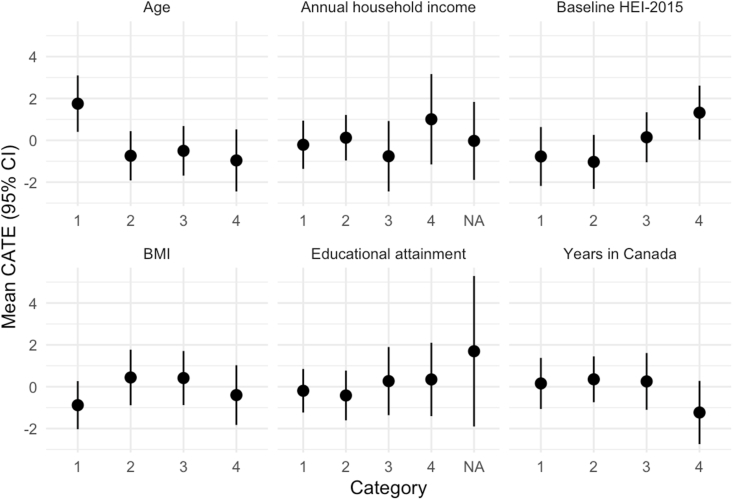
Mean conditional average treatment effects and 95% confidence intervals for the 6 most important variables identified in a causal forest. CATE, conditional average treatment effect; HEI-2015, Healthy Eating Index-2015.

On examining variable importance, BMI, baseline HEI-2015 score, age, the number of years lived in Canada, annual household income, and educational attainment emerged as the top 6 variables used in tree splits of the 12 variables included in the main causal forest (Table 3). Plots of the predicted CATEs of the 6 most important variables showed that most 95% CIs overlapped and crossed zero, indicating considerable uncertainty in estimated treatment effects (Figure 3).

**TABLE 3:** The 6 most important variables in identifying treatment effect heterogeneity identified using variable importance measures from the causal forest.

Rank	Variable	Variable importance measure
1	BMI	0.263
2	Baseline Healthy Eating Index-2015 score	0.198
3	Age	0.174
4	Years lived in Canada	0.132
5	Annual household Income	0.061
6	Educational attainment	0.054

### Traditional linear regression models

Interaction terms between the treatment and the 2 most important variables (BMI and baseline HEI-2015 score) in the linear regression models were not significant and had wide CIs, indicating no evidence of effect modification (Table 4). These findings align with those from the causal forest, with both approaches providing imprecise estimates and failing to detect heterogeneous treatment effects.

**TABLE 4:** Effect modification of FMNCP treatment effects on Healthy Eating Index-2015 scores (*n* = 263).

	β	95% CI	*P*
Group × BMI × baseline HEI-2015	–0.01	–0.06, 0.04	0.65
Group × BMI	0.42	–0.15, 1.00	0.15
Group × baseline HEI-2015	0.005	–0.24, 0.25	0.97

Linear regression models included group, sex, geographical location, age, highest educational attainment, race/ethnicity, marital status, smoking status, perceived physical health, number of household members, children living in the home, pregnancy, and breastfeeding.

Abbreviations: CI, confidence interval; FMNCP, Farmers’ Market Nutrition Coupon Program; HEI-2015, Healthy Eating Index-2015.

### Sensitivity analyses

Following all sensitivity analyses, the results from the main analysis remained unchanged and are detailed in Supplemental Figures 5–14. However, when the severity of household food insecurity was added as a predictor in the causal forest, although there were no heterogeneous treatment effects, it emerged as one of the 6 most important variables. Therefore, a causal forest was retrained with the 6 most important variables, which included household food insecurity (i.e., BMI, baseline HEI-2015 score, age, years lived in Canada, annual household income, and household food insecurity), and findings remained unchanged (data not shown).

### Simulation-based power analysis

**FIGURE 4:**
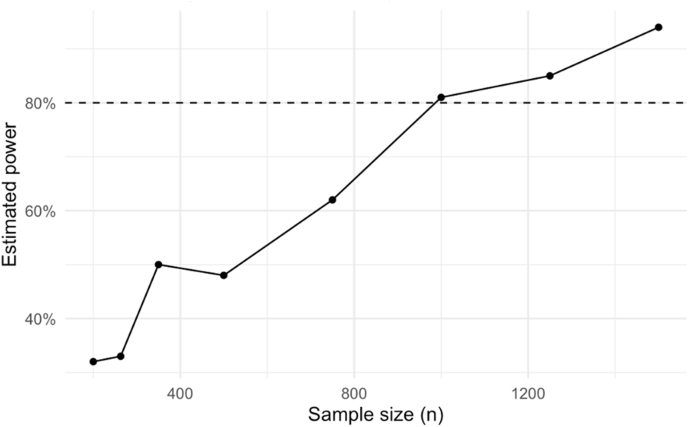
Power to detect a simulated 5-point difference in Healthy Eating Index-2015 scores between participants with low and high educational attainment using a causal forest.

**FIGURE 5:**
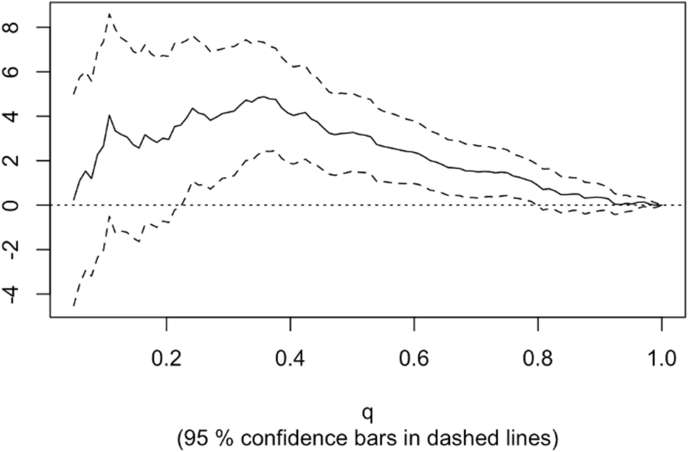
Targeting operator characteristic curve with 95% pointwise confidence intervals using a simulated sample of *n* = 1050 with a 5-point difference in Healthy Eating Index-2015 scores between participants with low and high educational attainment. Note: *q* = fraction treated.

The simulation-based power analysis showed that the sample size of 263 participants provided 40% power to detect the simulated heterogeneous treatment effects (Figure 4). A sample size of ∼1050 participants was required to achieve 80% power. At a simulated sample size of *n =* 1050, the best linear predictor test showed strong calibration (*α* = 0.99, *P* < 0.001) and detected the simulated heterogeneity in HEI-2015 scores (*β* = 1.11, *P* < 0.001) (Table 5). The TOC curve was above zero, and the AUTOC was positive and significant [2.25 (*P* = 0.003)], indicating that the causal forest successfully ranked participants according to their predicted CATEs and detected treatment effect heterogeneity with a sample size of 1050 (Figure 5).

**TABLE 5:** Best linear predictor test estimates from a causal forest using a simulated sample of *n* = 1050 with a 5-point difference in Healthy Eating Index-2015 scores between participants with low and high educational attainment.

	Estimate	*P* value
α (mean forest prediction)	0.99	<0.001
*β* (differential forest prediction)	1.11	<0.001

## Discussion

This study employed a causal forest analysis to examine heterogeneous treatment effects of the BC FMNCP on the diet quality of adults with low incomes and evaluated model performance in the context of a moderately sized pragmatic RCT. The causal forest did not detect heterogeneous treatment effects in HEI-2015 scores postintervention among participants in the BC FMNCP. Notably, the simulation-based power analysis indicated that the study was underpowered to detect simulated heterogeneous treatment effects. Overall, these results highlight key considerations when interpreting nonsignificant heterogeneous treatment effects and for applying and evaluating causal forests in moderately sized RCTs.

The causal forest did not detect heterogeneous treatment effects; however, diagnostic indicators including the nonsignificant best linear predictor test, flat TOC curve, negative and nonsignificant AUTOC, and wide CATE CIs, suggested limited power and substantial uncertainty in effect estimates rather than the absence of heterogeneity. The simulation-based power analysis further supports this interpretation, indicating that the study was underpowered, with ∼1050 participants required to achieve 80% power. In this context, careful evaluation of model performance is essential, and null findings should be interpreted with caution.

BMI, HEI-2015 score, age, years lived in Canada, annual household income, educational attainment, and household food insecurity severity emerged as the most important variables in growing the causal forest. However, visual inspection of CATE distributions for these variables showed substantial uncertainty, with wide, overlapping CIs. As such, variable importance measures should be viewed as exploratory and used to generate hypotheses rather than as evidence of clinically meaningful effect modifiers in moderately sized trials such as ours.

The limited power and uncertainty of effect estimates in this study suggest that causal forests may not outperform traditional methods for identifying treatment effect heterogeneity in moderately sized studies, as both regression-based approaches and the causal forest failed to detect heterogeneity and produced imprecise estimates. Prior studies have similarly found that causal forests do not consistently outperform regression-based approaches in detecting heterogeneous treatment effects [[67], [68], [69]]. However, causal forests may still serve as a valuable complement to traditional regression-based subgroup analyses by capturing nonlinear, higher-order interactions, particularly when sample sizes are larger [35,70]. In addition, causal forests offer a distinct advantage over traditional regression analyses and most machine learning approaches by estimating causal CATEs with valid CIs, rather than solely predicting outcomes.

Informed by these findings, several recommendations emerge for using causal forests to examine heterogeneous treatment effects in moderately sized trials. First, traditional regression-based subgroup analyses remain a sound starting point, as they provide interpretable tests of effect modification with well-established methodological properties [27]. Second, combining traditional subgroup analyses with causal forests can strengthen interpretation by showing whether both approaches point to similar conclusions. Third, in moderately sized samples, causal forest analyses should be accompanied by diagnostic checks, such as calibration tests, power-based simulations, and assessing leaf sample sizes to detect small-sample bias. Finally, nonsignificant heterogeneous treatment effects should not be interpreted as evidence that heterogeneity was absent. Smaller samples can lead to inadequately powered, high-variance treatment effect estimates, limiting the ability of both machine learning and traditional approaches to detect meaningful subgroup differences. Overall, application of causal forests in moderately sized trials can be useful for examining heterogeneous treatment effects, but should be interpreted as exploratory and supported by thorough diagnostic and power analyses.

In addition to serving as a case example of interpreting and evaluating nonsignificant heterogeneous treatment effects from a causal forest analysis, this study also summarizes practical guidance for applying causal forests in moderately sized trials. Specifically, we describe key design and analytic considerations, including model configuration (e.g., number of trees and tuning procedures), predictor selection and encoding, testing model assumptions, and diagnostic tools (e.g., calibration tests, TOC curves, and simulation-based power analyses) to assess model performance. We refer readers to several publications and online resources that provide more detailed guidance on using causal forests [19,28,32,50,71,72]. Overall, this study offers a structured approach for implementing and evaluating causal forests in settings where sample size constraints may limit the ability to detect heterogeneous treatment effects.

Some limitations of our analyses should be considered. We used a large number of trees in our main analysis. However, although larger forests can increase computational burden, they generally stabilize predictions rather than introduce bias [36,73]. Notably, fitting the causal forest with 11,000 trees was computationally feasible and did not meaningfully extend processing time, suggesting that increasing the number of trees to improve estimate stability may be practical even in moderately sized datasets. Given that our analysis was fundamentally limited by sample size, the number of trees used was unlikely to have materially influenced the results. In addition, we made several a priori decisions regarding predictor inclusion and coding, which may have influenced our findings [74]. However, conclusions from our main analysis remained unchanged when additional predictors were included and coded in various ways in sensitivity analyses. We also aimed to include key predictors of treatment effect heterogeneity; however, impacts on diet quality may have varied by other predictors that we did not include in our models. Moreover, heterogeneous treatment effects may not have been detected among smaller, underrepresented subgroups (e.g., males), and our null findings should be interpreted in this context. Additionally, caution is warranted when interpreting variable importance measures as they have important limitations. For example, causal forests tend to randomly select correlated variables for splits and prioritize continuous variables and categorical variables with a larger number of categories [71,75,76]. Finally, dietary intakes were self-reported and thus subject to misreporting [41]. However, 24-h dietary recalls tend to have less systematic bias than other self-report methods, such as food frequency questionnaires [77,78].

To our knowledge, this is the first study to employ and critically evaluate a causal forest to examine heterogeneous treatment effects in diet quality among adults with low incomes receiving a healthy food subsidy. Examining heterogeneous treatment effects can contribute to precision public health initiatives by helping to more precisely identify intersecting social structures that shape dietary inequities [21]. This information can then be used to identify specific points of intervention to reduce dietary inequities [21]. This study was underpowered to detect heterogeneous treatment effects, and thus our null findings should be interpreted with caution, as the limited statistical power precludes conclusions about whether meaningful subgroup differences exist. Larger datasets will be essential to reliably detect heterogeneous treatment effects and to realize the full potential of causal forests in public health nutrition research.

In conclusion, a causal forest did not detect heterogeneous treatment effects on diet quality among participants in the BC FMNCP. Simulation results indicated that the trial was underpowered to estimate heterogeneous treatment effects using causal forests, underscoring the need for larger trials. Nonetheless, with careful application and evaluation, causal forests remain a useful tool to explore heterogeneous treatment effects in moderately sized trials when the sample size is adequate. Although the causal forest did not detect participant subgroups who differentially benefited from the program, examining heterogeneous treatment effects remains essential for uncovering how intersecting social structures shape dietary inequities and identifying specific points of intervention to reduce them.

## Author contributions

The authors’ responsibilities were as follows – MLA: wrote the manuscript; MLA, SD: collected the data; MLA, IL, YC: conducted the statistical analyses; MLA, IL, YC, ZS, DLO: contributed to study design; and all authors: interpreted the data and critically edited and approved the final manuscript.

## Data availability

The datasets used and/or analyzed during the current study are available from the corresponding author on reasonable request.

## Funding

The authors reported no funding received for this study.

## Declaration of generative AI and AI-assisted technologies in the writing process

During the preparation of this work, the authors used ChatGPT-5 to improve language clarity and readability. After using this tool, the authors reviewed and edited the content as needed and take full responsibility for the content of the publication.

## Conflict of interest

HO is the Executive Director of the British Columbia Association of Farmers' Markets. PL is the Program Manager for the British Columbia Farmers’ Market Nutrition Coupon Program. All other authors report no conflicts of interest.
