# Supplementary material for: Causal Forests in Practice: Lessons on Detecting Heterogeneous Treatment Effects in a Randomized Controlled Trial of a Healthy Food Subsidy Program in Canada
Source: J Nutr. 2026 Apr 8;156(6):101529. doi: 10.1016/j.tjnut.2026.101529 (PMC13279299; doi:10.1016/j.tjnut.2026.101529)
Supplement: multimedia component 1 [file mmc1.docx]

**Supplemental materials**

**Leveraging machine learning in a randomized controlled trial: Heterogeneous treatment effects of the British Columbia Farmers’ Market Nutrition Coupon Program on diet quality**

Michelle L. Aktary, Inara Lalani, Yong Chen, Zahra Shakeri, Gavin R. McCormack, Sharlette Dunn, Tolulope Sajobi, Heather O'Hara, Peter Leblanc, Jenny Godley, Natalie Doan, Dana Lee Olstad

1. **Selection of baseline predictor variables**

Baseline candidate predictor variables were selected based on 1) the most important variables identified in a preliminary causal forest (**Supplemental Table 1**), 2) Pearson correlation coefficients between baseline predictors and HEI-2015 scores post-intervention (**Supplemental Table 2**), and 3) sociodemographic characteristics and health practices associated with diet quality identified in the published literature (**Supplemental Table 3**).

***Preliminary causal forest***

Using the variable importance measure, a preliminary causal forest was trained with 16 baseline variables to identify the variables that contributed most to tree splits. The causal forest was generated with 11,000 trees and used default model parameters included in *grf*. The cross-validation procedure was enabled, allowing model parameters (e.g., the number of variables tried for each split) to be tuned to improve the precision of estimates (1-3).

Baseline variables included age (years), sex (male, female), geographic location (urban, rural), body mass index (BMI), baseline Healthy Eating Index 2015 (HEI-2015) scores, pregnancy status (yes, no), breastfeeding (yes, no), years lived in Canada, self-reported physical health, number of household members, marital status (living with partner, not living with partner), current smoking status (yes, no), children living in the home (yes, no), race/ethnicity (White, racial/ethnic minority group), educational attainment (high school diploma or less, some post-secondary or trade, Bachelor's degree, Graduate degree), and annual household income before taxes (less than CAD $20,000, $20,000 to $39,999, $40,000 to $59,999, more than $60,000).

**Supplemental Table 1**. The 10 most important variables identified using preliminary causal forest variable importance measures

| Rank | Variable | Variable importance measure |
| --- | --- | --- |
| 1 | Body mass index | 0.239 |
| 2 | Age | 0.152 |
| 3 | Baseline Healthy Eating Index-2015 score | 0.147 |
| 4 | Years lived in Canada | 0.145 |
| 5 | Annual household Income | 0.126 |
| 6 | Educational attainment | 0.077 |
| 7 | Number of household members | 0.026 |
| 8 | Marital status | 0.023 |
| 9 | Children living in the home | 0.023 |
| 10 | Race/ethnicity | 0.021 |

***Pearson correlation coefficients***

Pearson correlation was used to examine correlations between baseline predictors and HEI-2015 scores post-intervention to help identify candidate predictor variables to include in the causal forest (**Supplemental Table 2**).

**Supplemental Table 2**. Pearson correlation coefficients between baseline sociodemographic characteristics and Healthy Eating Index-2015 scores at post-intervention

| **Baseline variables** | **Pearson correlation coefficient** | **p-value** |
| --- | --- | --- |
| Age | 0.101 | 0.104 |
| Sex | 0.042 | 0.495 |
| Geography (urban/rural) | 0.018 | 0.770 |
| Healthy Eating Index-2015 score | 0.464 | **<0.001** |
| Pregnant | 0.059 | 0.343 |
| Breastfeeding | 0.005 | 0.930 |
| Years lived in Canada | -0.159 | **0.010** |
| Body mass index | -0.111 | 0.077 |
| Number of household members | -0.001 | 0.982 |
| Children living in the home | -0.055 | 0.376 |
| Educational attainment | 0.184 | **0.003** |
| Annual household income | 0.008 | 0.907 |
| Marital status | 0.154 | **0.013** |
| Race/ethnicity | -0.159 | **0.014** |
| Smoking status | -0.272 | **<0.001** |
| Self-reported physical health | 0.048 | 0.437 |

***Sociodemographic characteristics associated with diet quality in the published literature***

It is well established that in Canada and other high-income countries, inequities in diet quality persist, and have even worsened in some cases (4, 5). For instance, in Canada and the United States (US), adults with lower incomes and educational attainment have poorer diet quality than their more advantaged counterparts (4, 5). Diet quality also varies by race/ethnicity. In Canada, recent evidence suggests that individuals from most racial/ethnic minority groups (e.g., those who identify as Black or South Asian) have similar or higher diet quality than White adults, whereas Indigenous adults have lower diet quality (6). In the US, non-Hispanic Black adults have the highest prevalence of poor diet quality compared to other racial/ethnic groups, whereas the prevalence of poor diet quality among Hispanic and non-Hispanic Asian adults is lower than that of non-Hispanic White adults (5). Diet quality is also influenced by the number of years lived in Canada. Immigrants new to Canada tend to have higher diet quality than native-born Canadians (7); however, their diet quality generally declines over time due to dietary acculturation, whereby immigrants adopt the dietary patterns of their host country (8, 9). In addition, diet quality tends to be higher among females (5, 7, 10-13), older adults (5, 11, 13, 14), and non-smokers (7, 11, 13, 14). Studies have also shown inverse associations between higher diet quality and obesity-related outcomes (e.g., BMI and waist circumference) (13-15). Pregnancy and breastfeeding (16, 17) and self-perceived health (18) have also been shown to shape diet quality.

Research on the association between geographic location (rural versus urban) and diet quality has shown mixed findings and suggests that diet quality among residents is influenced by a variety of factors, such as socioeconomic position and neighbourhood density (19-21). However, in a qualitative study of the British Columbia Farmers’ Market Nutrition Coupon Program, participants indicated that the variety of foods available at rural farmers’ markets was more limited than those from urban farmers’ markets (22). Fewer healthful food choices from farmers’ markets may limit program impacts on diet quality. Previous studies have also shown that household composition shapes dietary patterns and purchases (23, 24). For instance, single-person households are less likely to purchase fruits and vegetables than larger households (24). Lone mothers experiencing food insecurity also tend to compromise their dietary intake to ensure their children have sufficient food (25). Finally, baseline diet quality has been shown to predict changes in diet quality, with changes more likely among individuals with the lowest baseline diet quality (26).

***Final selection of candidate predictors at baseline***

Based on the above three considerations, variables that met at least two of the three criteria were included in the main causal forest analysis. These variables included age, smoking status, race/ethnicity, annual household income, educational attainment, years lived in Canada, BMI, marital status, and baseline HEI-2015 scores (**Supplemental Table 3**). Although children living in the home and number of household members also met two of the three criteria, we only included children living in the home to reduce the number of variables in the causal forest. We chose children living in the home over number of household members, given the evidence that parents compromise their own dietary intakes to prioritize children’s food and nutrient intake in an effort to shield them from experiences of household food insecurity (25). While sex met only one criterion, it was included because it is a key determinant of diet quality (14, 27). Geographic location was also included, as FMNCP participants reported limited food options in rural farmers’ markets (22); thus, it was considered a potentially important predictor of treatment effect heterogeneity.

**Supplemental Table 3**. Baseline predictor variables selected for the main causal forest analysis

| **Baseline variables** | **Variable importance measure** | **Pearson correlation coefficient** | **Literature** | **Included in main analysis** |
| --- | --- | --- | --- | --- |
| Age | ✓ |  | ✓ | ✓ |
| Sex |  |  | ✓ | ✓ |
| Geography (urban/rural) |  |  | ✓ | ✓ |
| Healthy Eating Index-2015 score | ✓ | ✓ | ✓ | ✓ |
| Pregnant |  |  | ✓ |  |
| Breastfeeding |  |  | ✓ |  |
| Years lived in Canada | ✓ | ✓ | ✓ | ✓ |
| Body mass index | ✓ |  | ✓ | ✓ |
| Number of household members | ✓ |  | ✓ |  |
| Children living in the home | ✓ |  | ✓ | ✓ |
| Educational attainment | ✓ | ✓ | ✓ | ✓ |
| Annual household income | ✓ |  | ✓ | ✓ |
| Marital status | ✓ | ✓ | ✓ | ✓ |
| Race/ethnicity | ✓ | ✓ | ✓ | ✓ |
| Smoking status |  | ✓ | ✓ | ✓ |
| Self-reported physical health |  |  | ✓ |  |

1. **Variance estimates with varying number of trees**

A causal forest was fit with 11,000 trees, the number of trees at which the mean variance of treatment effect estimates was minimized (**Supplemental Figure 1)**.


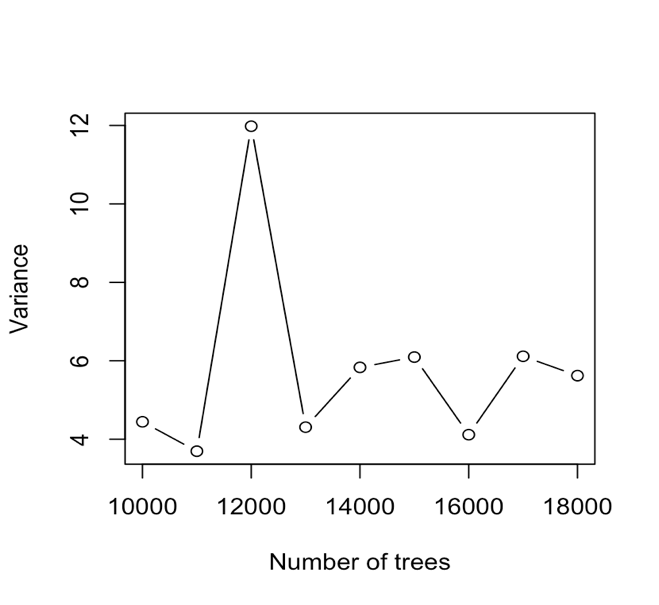


**Supplemental Figure 1**. Mean variance of conditional average treatment effect (CATE) estimates by number of trees in a causal forest

1. **Model assumptions**

Estimating conditional average treatment effects (CATEs) requires that two assumptions are met. The unconfoundedness assumption (i.e., conditional treatment assignment is independent of potential outcomes) and the overlap assumption (i.e., there are individuals in the treatment and control groups with all values of observable covariates) are generally fulfilled in randomized controlled trials (RCTs) (3, 28-30). While unconfoundedness is untestable because it assumes the determinants of treatment assignment are independent of potential outcomes (3, 28-30), the overlap assumption was evaluated using a propensity score histogram. The propensity score is the probability of a participant being assigned to the treatment group (30). In RCTs with two groups, each participant has a 50% chance of being assigned to the treatment group, yielding a propensity score of 0.50. **Supplemental Figure 2** shows that the propensity scores are centred around 0.5, indicating that participants were equally likely to be in the intervention or control group, and thus the overlap assumption was met.


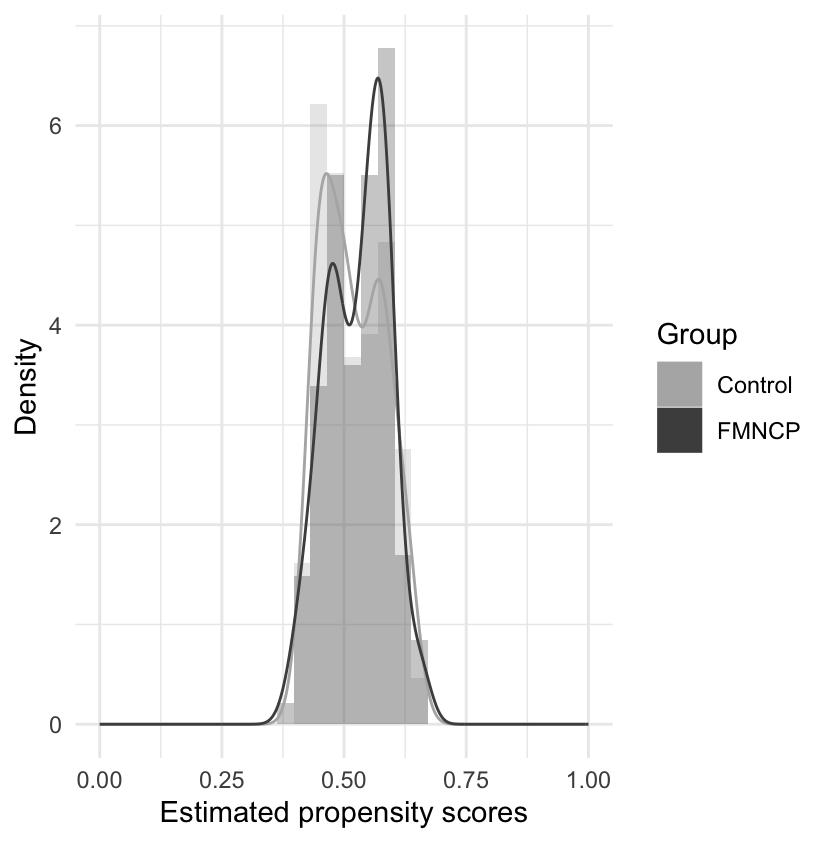


**Supplemental Figure 2**. Histogram of the causal forest estimated propensity scores of control and intervention groups

1. **Model fit**

Model fit was assessed using subsample validation (31). Subsamples were drawn from the test dataset and mean CATEs were predicted over 20 iterations. For each iteration, a histogram was constructed to examine the distribution of mean CATEs, which varied slightly but overall remained fairly uniform (**Supplemental Figure 3**). The mean CATE of each iteration was then plotted, which showed small variability in the predictions (**Supplemental Figure 4**). Overall, **Supplemental Figures 3 and 4** show stable estimates across iterations, suggesting adequate model fit. Finally, an analysis of variance tested for differences in mean CATEs in subsamples across the 20 iterations and showed no significant differences (p=1.0), further suggesting no evidence of overfitting.


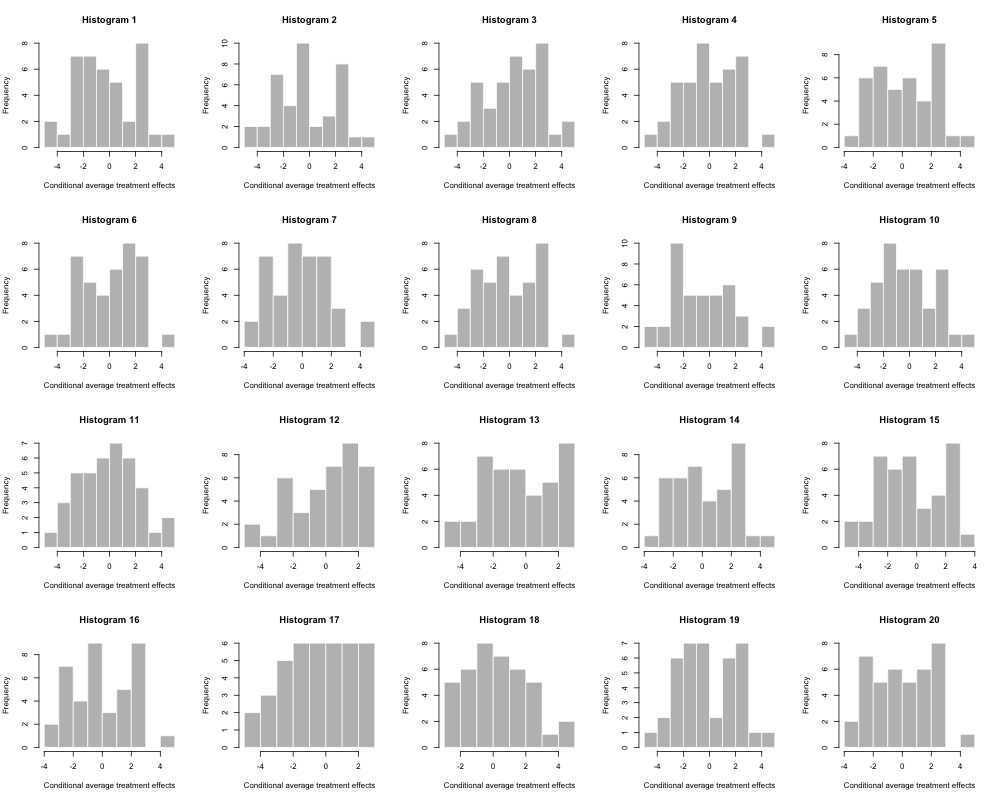


**Supplemental Figure 3**. Histograms of mean conditional average treatment effects predicted across 20 iterations from subsamples drawn from the test set


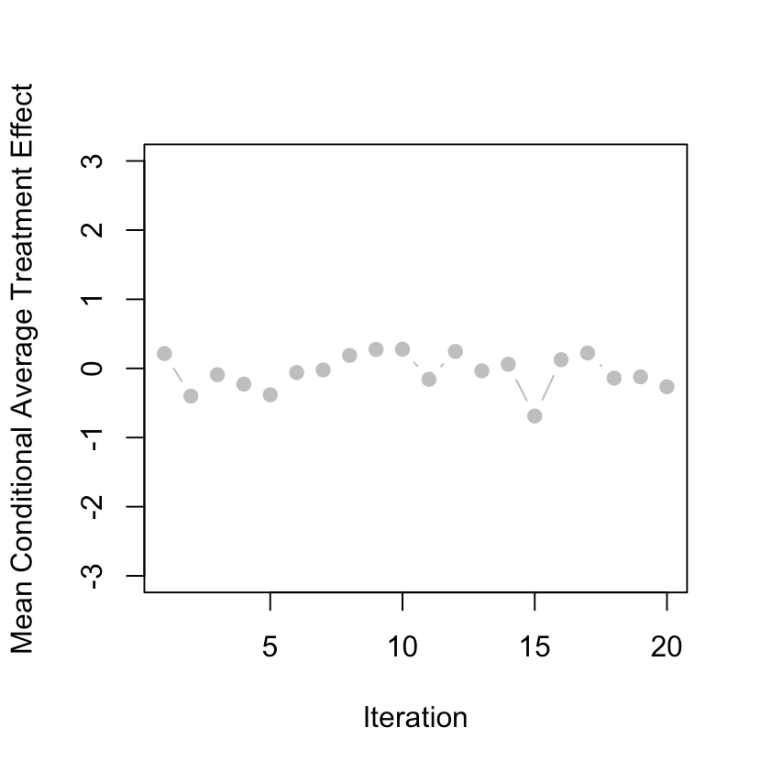


**Supplemental Figure 4**. Plotted mean conditional average treatment effect predictions of subsamples across 20 iterations

1. **Sensitivity analyses**

***Multiple Imputation by chained equations***

Missing baseline covariates and HEI-2015 scores at post-intervention were imputed using multiple imputation by chained equations. The estimated ATE was -0.89 (95% CI -2.22, 0.44), aligning with findings from our main analysis. The histogram of the pooled CATES is presented in **Supplemental Figure 5** and shows a similar distribution to the main analysis. The best linear predictor test did not detect heterogeneity, with a pooled differential forest prediction of 0.67. Likewise, the AUTOC was slightly higher than that from the main analysis but remained non-significant (2.74 (p=0.28)), and the TOC curve remained relatively flat (**Supplemental Figure 6**).

**
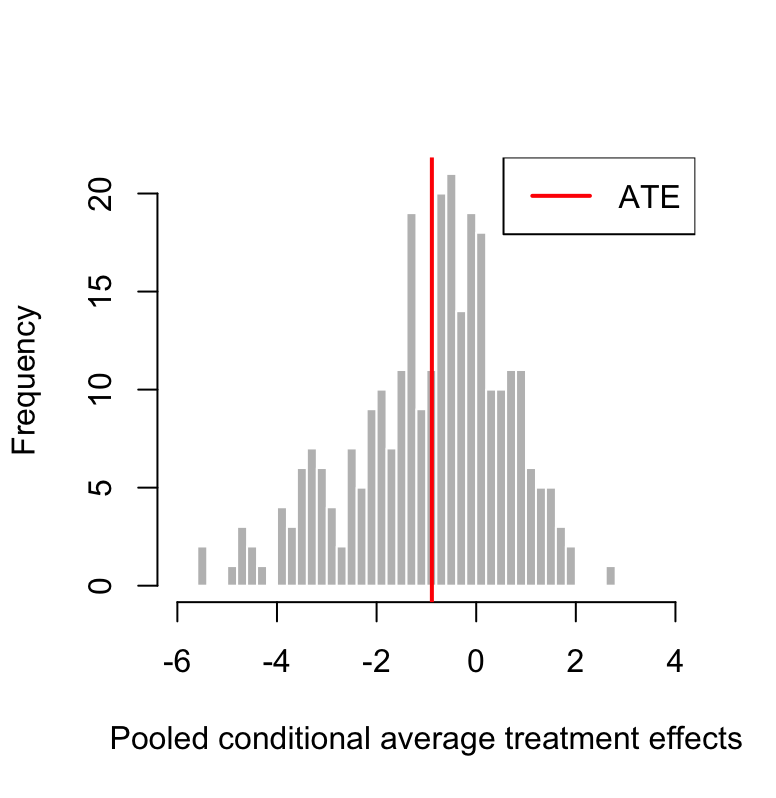
**

**Supplemental Figure 5.** Histogram of the pooled average treatment effects (ATE) and conditional average treatment effects derived from a causal forest using imputed data


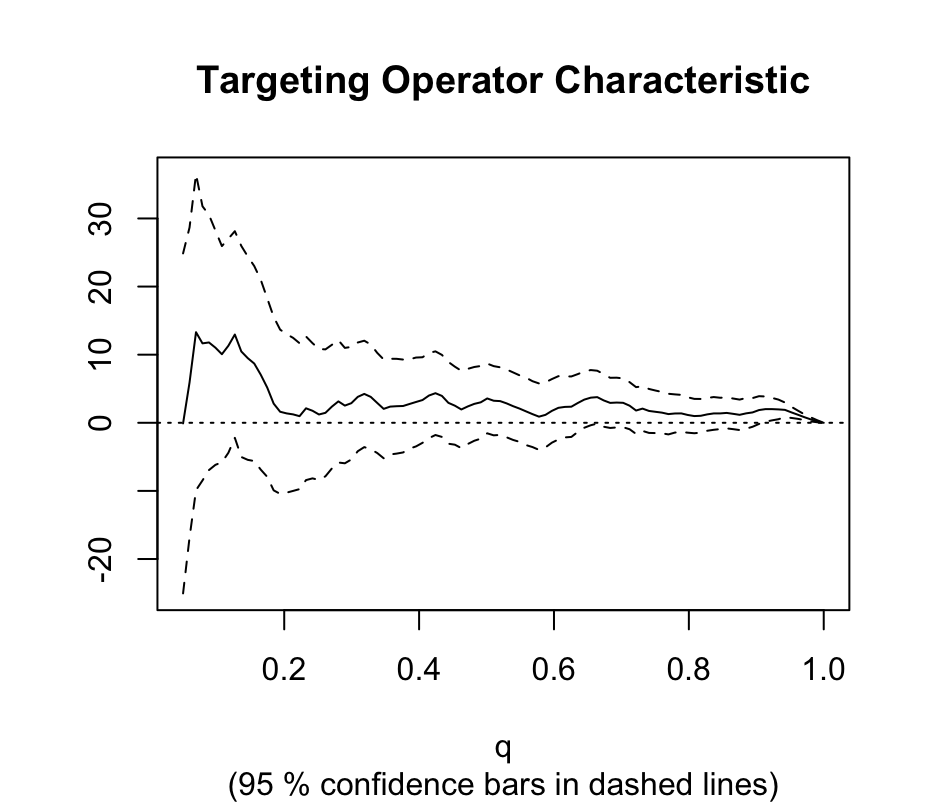


**Supplemental Figure 6**. Targeting operator characteristic curve for predicted conditional average treatment effects and pointwise 95% confidence intervals (dashed lines) derived from a causal forest using imputed data

Note: q = fraction treated

***Causal forest trained with the six most important variables***

A causal forest was trained using only the six most important variables (BMI, baseline HEI-2015 score, age, years lived in Canada, annual household income, and educational attainment) identified in the main analysis. The estimated ATE was -0.89 (95% CI -3.98, 2.19), and the histogram of the CATEs showed a narrower distribution of CATEs than the main analysis (**Supplemental Figure 7**). The best linear predictor test did not detect heterogeneous treatment effects, with a differential forest prediction of 0.40 (p=0.35), which was similar to the main analysis. The TOC curve remained relatively flat, and the estimated AUTOC of 0.96 (p=0.73) was non-significant (**Supplemental Figure 8)**.


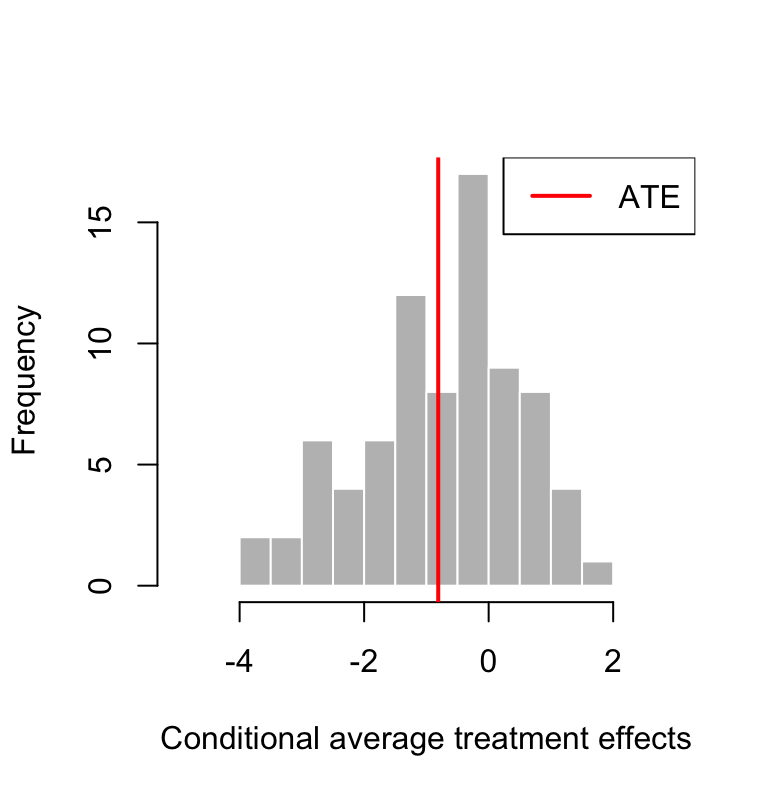


**Supplemental Figure 7**. Histogram of the average treatment effects (ATE) and conditional average treatment effects derived from a causal forest trained with the six most important variables (BMI, baseline HEI-2015 score, age, years lived in Canada, annual household income, and educational attainment)


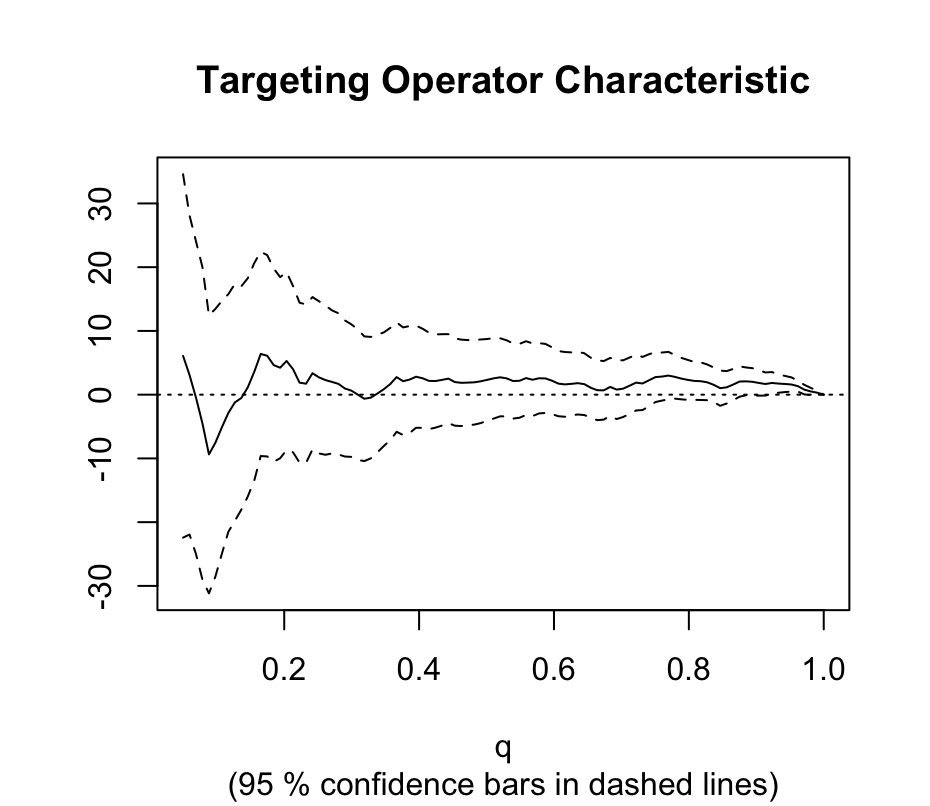


**Supplemental Figure 8**. Targeting operator characteristic curve for predicted conditional average treatment effects and pointwise 95% confidence intervals (dashed lines) derived from a causal forest trained with the six most important variables (BMI, baseline HEI-2015 score, age, years lived in Canada, annual household income, and educational attainment)

Note: q = fraction treated

***Causal forest trained with new or recoded variables***

No heterogenous treatment effects were detected when the variable children living in the home (yes, no) was replaced with the number of children living in the home (1, 2, 3, 4+) and separate variables for each racial/ethnic group (White, East and Southeast Asian, South and West Asian, Indigenous, and all other racial/ethnic groups) were included in a causal forest. The ATE was -0.64 (95% CI -3.76, 2.48); however, the distribution of CATEs was wider than the main analysis and shifted more towards positive values (**Supplemental Figure 9**). Similarly, the best linear predictor test (differential forest prediction 0.28 (p=0.35)) and the estimated AUTOC (0.92 (p=0.73)) were non-significant. The TOC curve remained relatively flat (**Supplemental Figure 10**).

Similar findings were observed when the number of children living in the home and separate variables for Indigenous status and racial/ethnic group were included in a causal forest. The ATE was -0.82 (95% CI -3.96, 2.32), with a narrower distribution of CATEs than the main analysis (**Supplemental Figure 11)**. The differential forest prediction from the best linear predictor test remained relatively unchanged (0.45, p=0.29). The estimated AUTOC was slightly higher than that from the main analysis but remained non-significant (1.57 (p=0.59)), and the TOC remained relatively flat (**Supplemental Figure 12**).


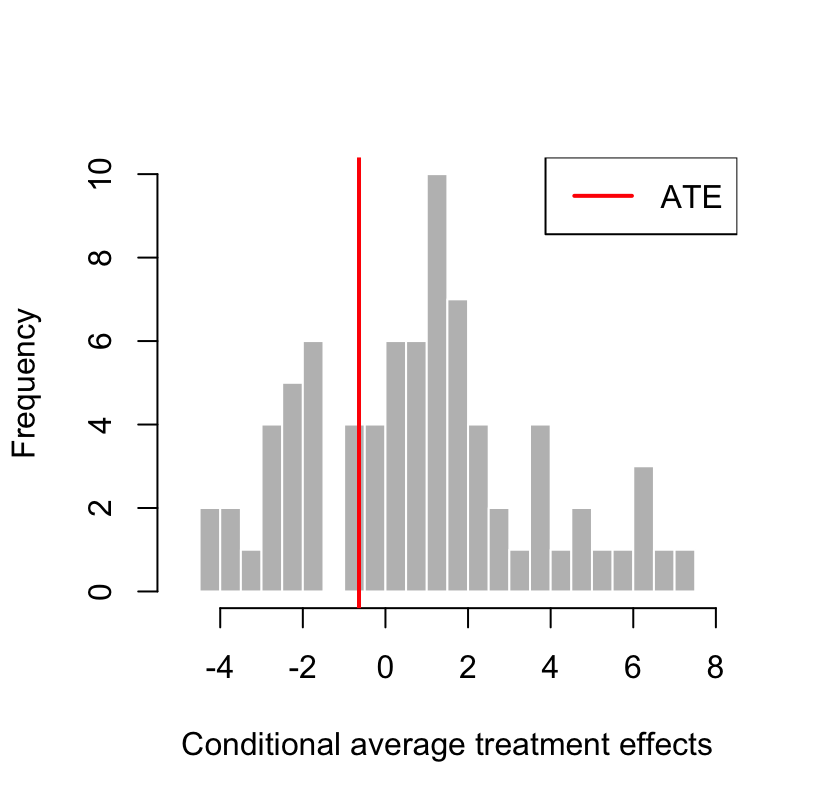


**Supplemental Figure 9.** Histogram of the average treatment effects (ATE) and conditional average treatment effects. The variable children living in the home was recoded from yes, no to 1, 2, 3, 4+, and race/ethnicity (White, Racial/ethnic minority group) was replaced with a separate variable for each racial/ethnic group (White, East and Southeast Asian, South and West Asian, Indigenous, and all other racial/ethnic groups)


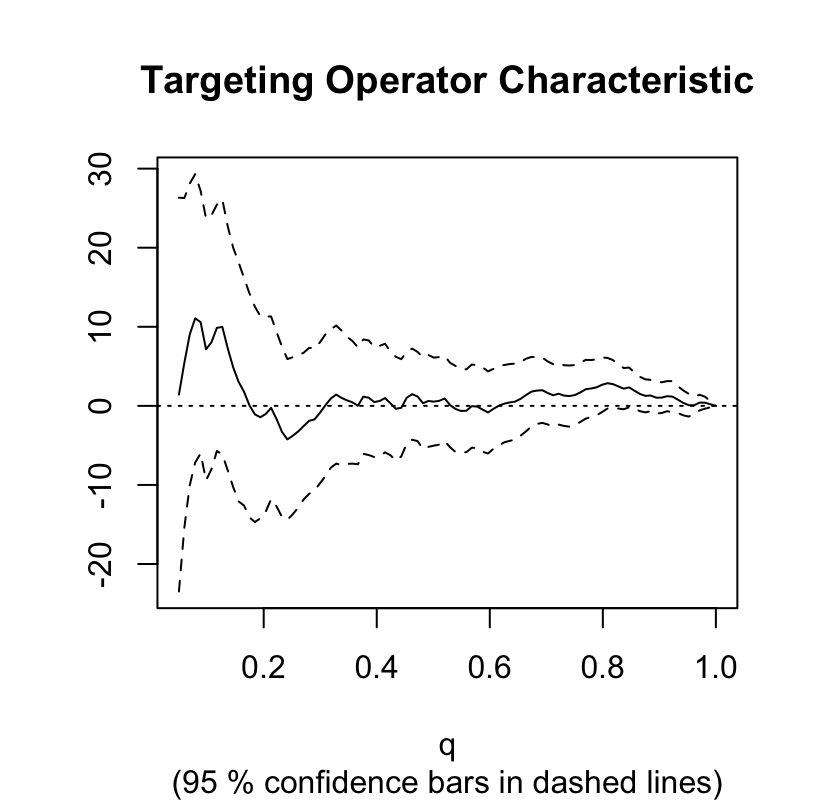


**Supplemental Figure 10**. Targeting operator characteristic curve for predicted conditional average treatment effects and pointwise 95% confidence intervals (dashed lines). The variable children living in the home was recoded from yes, no to 1, 2, 3, 4+, and race/ethnicity (White, Racial/ethnic minority group) was replaced with a separate variable for each racial/ethnic group (White, East and Southeast Asian, South and West Asian, Indigenous, and all other racial/ethnic groups)

Note: q = fraction treated


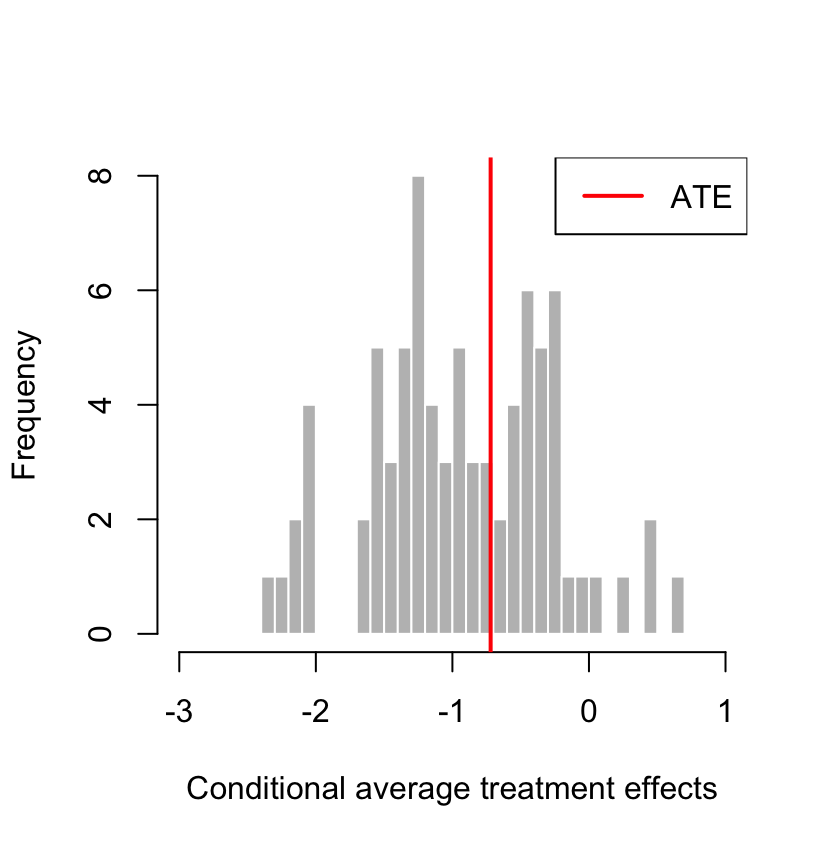


**Supplemental Figure 11.** Histogram of the average treatment effects (ATE) and conditional average treatment effects. The variable children living in the home was recoded from yes, no to 1, 2, 3, 4+, and race/ethnicity (White, racial/ethnic minority group) was replaced with race/ethnicity (White, racial/ethnic minority group, excluding Indigenous peoples) and Indigenous status (Indigenous vs non-Indigenous)


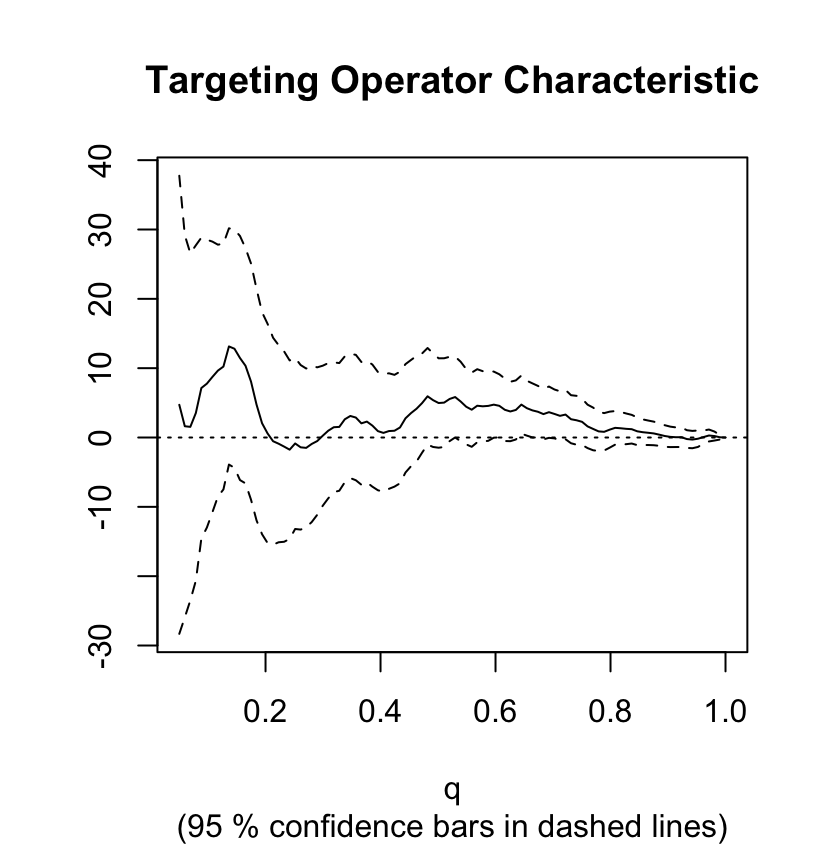


**Supplemental Figure 12**. Targeting operator characteristic curve for predicted conditional average treatment effects and pointwise 95% confidence intervals (dashed lines). The variable children living in the home was recoded from yes, no to 1, 2, 3, 4+, and race/ethnicity (White, racial/ethnic minority group) was replaced with race/ethnicity (White, racial/ethnic minority group, excluding Indigenous people) and Indigenous group (Indigenous vs non-Indigenous)

Note: q = fraction treated

***Causal forest trained with household food insecurity***

When baseline severity of household food insecurity (categorized as food secure, and marginal, moderate, and severe food insecurity) was added as a predictor variable in the causal forest, findings remained unchanged. The ATE was -0.85 (95% CI -3.97, 2.27); however, compared to the main analysis, the distribution of CATEs shifted more towards negative values (**Supplemental Figure 13**). The best linear prediction test showed a differential forest prediction of 0.50 (p=0.28). The AUTOC was higher than that from the main analysis but was non-significant (2.06 (p=0.39)), and the TOC remained relatively flat **Supplemental Figure 14**.


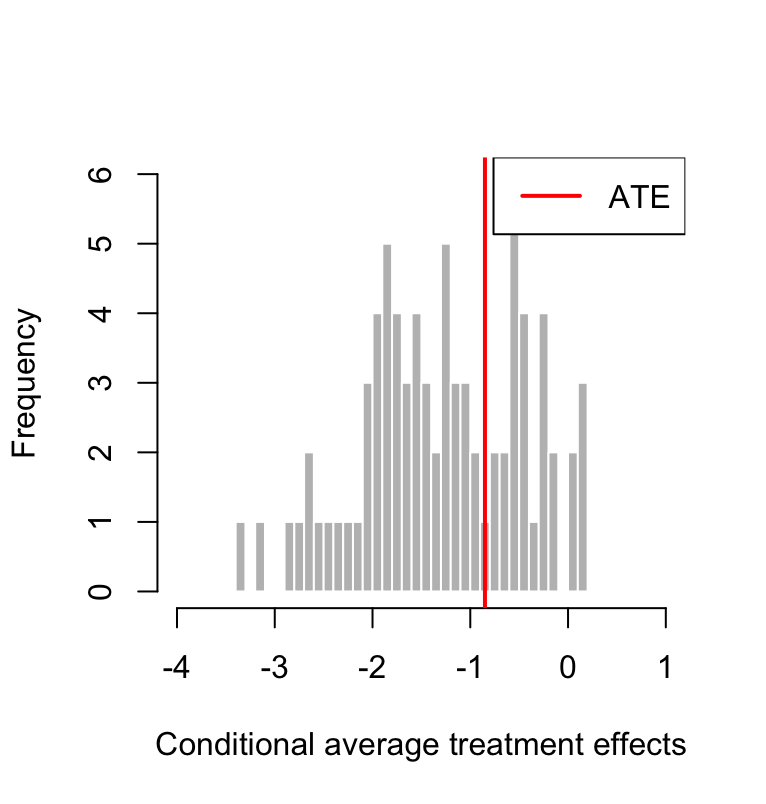


**Supplemental Figure 13**. Histogram of the average treatment effects (ATE) and conditional average treatment effects with severity of household food insecurity included as a predictor variable.


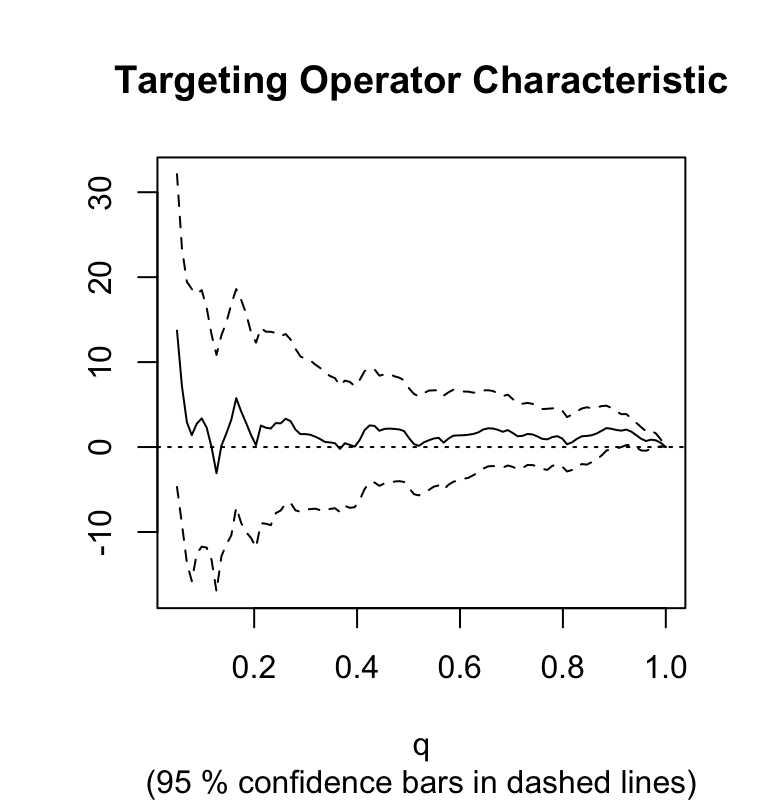


**Supplemental Figure 14**. Targeting operator characteristic curve for predicted conditional average treatment effects and pointwise 95% confidence intervals (dashed lines) derived from a causal forest that included severity of household food insecurity as a predictor variable

Note: q = fraction treated

**References**

1. Athey S, Imbens G. Recursive partitioning for heterogeneous causal effects. Proc Natl Acad Sci U S A. 2016;113(27):7353-60. Epub 2016/07/07. doi: 10.1073/pnas.1510489113. PubMed PMID: 27382149; PubMed Central PMCID: PMCPMC4941430.

2. Athey S, Tibshirani J, Wager S. Generalized random forests. Ann Stat. 2019;47:1148–78.

3. Athey S, Wager S. Estimating Treatment Effects with Causal Forests- An Application. Cornell University, 2019 Contract No.: arXiv:1902.07409 [stat.ME].

4. Olstad DL, Nejatinamini S, Victorino C, Kirkpatrick SI, Minaker LM, McLaren L. Socioeconomic inequities in diet quality among a nationally representative sample of adults living in Canada: an analysis of trends between 2004 and 2015. Am J Clin Nutr. 2021. Epub 2021/09/04. doi: 10.1093/ajcn/nqab249. PubMed PMID: 34477821.

5. Liu J, Mozaffarian D. Trends in Diet Quality Among U.S. Adults From 1999 to 2020 by Race, Ethnicity, and Socioeconomic Disadvantage. Ann Intern Med. 2024;177(7):841-50. Epub 2024/06/17. doi: 10.7326/M24-0190. PubMed PMID: 38885507.

6. Olstad DL, Nejatinamini S, Blanchet R, Moubarac JC, Polsky J, Vanderlee L, et al. Protecting traditional cultural food practices: Trends in diet quality and intake of ultra-processed foods by Indigenous status and race/ethnicity among a nationally representative sample of adults in Canada. SSM Popul Health. 2023;24:101496. Epub 2023/09/13. doi: 10.1016/j.ssmph.2023.101496. PubMed PMID: 37701069; PubMed Central PMCID: PMCPMC10493595.

7. Hosseini SH, Farag M, Hosseini SZ, Vatanparast H. Behavioral factors are perhaps more important than income in determining diet quality in Canada. SSM Popul Health. 2022;17:101001. Epub 2022/01/11. doi: 10.1016/j.ssmph.2021.101001. PubMed PMID: 35005185; PubMed Central PMCID: PMCPMC8715369.

8. Davison KM, Gondara L. A Comparison of Mental Health, Food Insecurity, and Diet Quality Indicators between Foreign-Born Immigrants of Canada and Native-Born Canadians. Journal of Hunger & Environmental Nutrition. 2019;16(1):109-32. doi: 10.1080/19320248.2019.1672601.

9. Sanou D, O'Reilly E, Ngnie-Teta I, Batal M, Mondain N, Andrew C, et al. Acculturation and nutritional health of immigrants in Canada: a scoping review. J Immigr Minor Health. 2014;16(1):24-34. Epub 2013/04/19. doi: 10.1007/s10903-013-9823-7. PubMed PMID: 23595263; PubMed Central PMCID: PMCPMC3895180.

10. Jessri M, Ng AP, L’Abbé MR. Adapting the Healthy Eating Index 2010 for the Canadian Population: Evidence from the Canadian Community Health Survey. Nutrients. 2017;9(8). doi: 10.3390/nu9080910.

11. Brassard D, Elvidge Munene LA, St-Pierre S, Guenther PM, Kirkpatrick SI, Slater J, et al. Evaluation of the Healthy Eating Food Index (HEFI)-2019 measuring adherence to Canada's Food Guide 2019 recommendations on healthy food choices. Appl Physiol Nutr Metab. 2022;47(5):595-610. Epub 2022/01/15. doi: 10.1139/apnm-2021-0415. PubMed PMID: 35030038.

12. Hiza HAB, Casavale KO, Guenther PM, Davis CA. Diet Quality of Americans Differs by Age, Sex, Race/Ethnicity, Income, and Education Level. J Acad Nutr Diet. 2013;113(2):297-306. doi: 10.1016/j.jand.2012.08.011.

13. Grech A, Sui Z, Siu HY, Zheng M, Allman-Farinelli M, Rangan A. Socio-Demographic Determinants of Diet Quality in Australian Adults Using the Validated Healthy Eating Index for Australian Adults (HEIFA-2013). Healthcare (Basel). 2017;5(1). Epub 2017/02/07. doi: 10.3390/healthcare5010007. PubMed PMID: 28165394; PubMed Central PMCID: PMCPMC5371913.

14. Kang M, Park SY, Shvetsov YB, Wilkens LR, Marchand LL, Boushey CJ, et al. Sex differences in sociodemographic and lifestyle factors associated with diet quality in a multiethnic population. Nutrition. 2019;66:147-52. Epub 2019/07/10. doi: 10.1016/j.nut.2018.11.022. PubMed PMID: 31288218; PubMed Central PMCID: PMCPMC7085987.

15. Asghari G, Mirmiran P, Yuzbashian E, Azizi F. A systematic review of diet quality indices in relation to obesity. Br J Nutr. 2017;117(8):1055-65. Epub 2017/05/10. doi: 10.1017/S0007114517000915. PubMed PMID: 28478768.

16. Doyle IM, Borrmann B, Grosser A, Razum O, Spallek J. Determinants of dietary patterns and diet quality during pregnancy: a systematic review with narrative synthesis. Public Health Nutr. 2017;20(6):1009-28. Epub 2016/11/18. doi: 10.1017/S1368980016002937. PubMed PMID: 27852338; PubMed Central PMCID: PMCPMC10261556.

17. Iacovou M, Gibson PR, Muir JG. Dietary Changes Among Breastfeeding Mothers. Journal of Human Lactation. 2021;37(3):566-76. doi: 10.1177/0890334420959283.

18. Collins CE, Young AF, Hodge A. Diet Quality Is Associated with Higher Nutrient Intake and Self-Rated Health in Mid-Aged Women. Journal of the American College of Nutrition. 2008;27(1):146-57. doi: 10.1080/07315724.2008.10719686.

19. Gilham K, Gu Q, Dummer TJB, Spinelli JJ, Murphy RA. Diet Quality and Neighborhood Environment in the Atlantic Partnership for Tomorrow's Health Project. Nutrients. 2020;12(10). Epub 2020/10/25. doi: 10.3390/nu12103217. PubMed PMID: 33096731; PubMed Central PMCID: PMCPMC7588981.

20. Martin JC, Moran LJ, Teede HJ, Ranasinha S, Lombard CB, Harrison CL. Exploring Diet Quality between Urban and Rural Dwelling Women of Reproductive Age. Nutrients. 2017;9(6). Epub 2017/06/09. doi: 10.3390/nu9060586. PubMed PMID: 28594351; PubMed Central PMCID: PMCPMC5490565.

21. Pullen R, Kent K, Sharman MJ, Schumacher TL, Brown LJ. A Comparison of Diet Quality in a Sample of Rural and Urban Australian Adults. Nutrients. 2021;13(11). Epub 2021/11/28. doi: 10.3390/nu13114130. PubMed PMID: 34836385; PubMed Central PMCID: PMCPMC8624345.

22. Caron-Roy S, Sayed SA, Milaney K, Lashewicz B, Dunn S, O'Hara H, et al. 'My coupons are like gold': experiences and perceived outcomes of low-income adults participating in the British Columbia Farmers' Market Nutrition Coupon Program. Public Health Nutr. 2021;25(2):1-12. Epub 2021/04/13. doi: 10.1017/S1368980021001567. PubMed PMID: 33843563.

23. Bukari M, Saaka M, Masahudu A, Ali Z, Abubakari AL, Danquah LO, et al. Household factors and gestational age predict diet quality of pregnant women. Matern Child Nutr. 2021;17(3):e13145. Epub 2021/02/03. doi: 10.1111/mcn.13145. PubMed PMID: 33528101; PubMed Central PMCID: PMCPMC8189244.

24. Silva A, Rivera M, Duran-Aguero S, Sactic MI. Single-Person Households: Insights from a Household Survey of Fruit and Vegetable Purchases. Nutrients. 2024;16(17). Epub 2024/09/14. doi: 10.3390/nu16172851. PubMed PMID: 39275169; PubMed Central PMCID: PMCPMC11397517.

25. McIntyre L, Glanville NT, Raine KD, Dayle JB, Anderson B, Battaglia N. Do low-income lone mothers compromise their nutrition to feed their children? JAMC. 2003;168(6):686-91.

26. Shatenstein B, Gauvin L, Keller H, Richard L, Gaudreau P, Giroux F, et al. Individual and collective factors predicting change in diet quality over 3 years in a subset of older men and women from the NuAge cohort. Eur J Nutr. 2016;55(4):1671-81. Epub 2015/07/15. doi: 10.1007/s00394-015-0986-y. PubMed PMID: 26169872.

27. Imamura F, Micha R, Khatibzadeh S, Fahimi S, Shi P, Powles J, et al. Dietary quality among men and women in 187 countries in 1990 and 2010: a systematic assessment. Lancet Glob Health. 2015;3(3):e132-42. Epub 2015/02/24. doi: 10.1016/S2214-109X(14)70381-X. PubMed PMID: 25701991; PubMed Central PMCID: PMCPMC4342410.

28. Green J, White II MH. Machine Learning for Experiments in the Social Sciences: Cambridge University Press; 2023.

29. Wager S, Athey S. Estimation and Inference of Heterogeneous Treatment Effects using Random Forests. Journal of the American Statistical Association. 2018;113(523):1228-42. doi: 10.1080/01621459.2017.1319839.

30. Sverdrup E, Petukhova M, Wager S. Estimating Treatment Effect Heterogeneity in Psychiatry- A Review and Tutorial with Causal Forests. Cornell University: 2024 Contract No.: arXiv:2409.01578 [stat.AP].

31. Kuhn M, Johnson K. Over-Fitting and Model Tuning. Applied Predictive Modeling. New York, NY: Springer; 2016. p. 61-92.
